# Editorial: Genomic insights on fungal hybrids

**DOI:** 10.3389/ffunb.2022.1063609

**Published:** 2022-11-07

**Authors:** Toni Gabaldón, Chris Todd Hittinger

**Affiliations:** ^1^Barcelona Supercomputing Centre (BSC-CNS). Plaça Eusebi Güell, Barcelona, Spain; ^2^Institute for Research in Biomedicine (IRB Barcelona), The Barcelona Institute of Science and Technology, Baldiri Reixac, Barcelona, Spain; ^3^Catalan Institution for Research and Advanced Studies (ICREA), Barcelona, Spain; ^4^Centro de Investigación Biomédica En Red de Enfermedades Infecciosas (CIBERINFEC), Barcelona, Spain; ^5^Laboratory of Genetics, DOE Great Lakes Bioenergy Research Center, J. F. Crow Institute for the Study of Evolution, Center for Genomic Science Innovation, Wisconsin Energy Institute, University of Wisconsin-Madison, Madison, WI, United States

**Keywords:** fungal hybrids, hybridization, introgression, polyploidy, genome

Hybrids are chimeric organisms that result from the crossing of two genetically divergent lineages. Compared to their parents, hybrids sometimes show higher adaptive capacities towards specific niches, thereby contributing to diversification (Abbott et al., 2013). Fungal hybrids have been neglected by formal studies for a long time due to the inherent challenges of the microbial species concept and the difficulty of identifying hybrids based on morphological characters (Gabaldón, 2020a; Boekhout et al., 2021)⁠. Although the first fungal hybrids were identified in *Saccharomyces*, thanks to careful dissection of metabolic properties (Morales and Dujon, 2012)⁠ and emerging genome sequencing technologies (Hittinger, 2013), it was the spread of these sequencing technologies that revealed the true pervasiveness of hybrids across the fungal tree of life (Naranjo-Ortiz and Gabaldón, 2020; Gabaldón, 2020b)⁠. Sequencing techniques, particularly genomic approaches, have not only unearthed the hybrid nature of many fungal organisms, but also serve as ideal tools for the study of hybrids. Hybrids have chimeric genomes, which usually display high instability and are subject to evolutionary pressures that are different from that of non-hybrid genomes (Runemark et al., 2019)⁠. Understanding how hybrids are formed, how they cope with their chimeric genomes, and how they evolve and adapt to distinct environments is a matter of intensive research. This Research Topic gathers six outstanding contributions that use genomic approaches to tackle diverse questions that relate to fungal hybrids.

One article by Ament-Velásquez et al. ⁠provides a compelling example of how genomics can reveal past hybridization, in this case in the lichen-forming *Letharia.* Guided by unexpected findings of triploid-like individuals, they used genomics to determine that these are hybrids between *L. lupina* and a so-far-undescribed *Letharia* lineage. Three other articles focus on the aftermath of hybridization at different time scales in the genus *Saccharomyces*. The article by Drouin et al. tests the genomic shock hypothesis using transposable element expression in artificial hybrids of different *Saccharomyces* species. The genomic shock hypothesis states that newly formed hybrids experience massive gene misregulation, including the activation of normally silenced transposable elements, due to incompatibilities of the two merged gene regulatory networks (McClintock, 1984)⁠. Contrary to expectations from this hypothesis, the Drouin et al. study shows that transposable elements are generally not differentially expressed in hybrids, even when exposed to stress conditions. This result adds to recent findings on the overall maintenance of gene expression patterns in yeast hybrids (Hovhannisyan et al., 2020a; Hovhannisyan et al., 2020b)⁠, and suggests that the genomic shock hypothesis, which was proposed based on observations of plant hybrids, may not hold for yeast hybrids. Another article from Bendixsen et al. focuses on genomic variation following hybridization by analyzing genomic data from over 200 naturally occurring hybrids between different species of the genus *Saccharomyces*⁠. The study reports common aneuploidies in hybrids, highlighting genome instability. Interestingly, their findings show that hybrids from more divergent parents result in more similar hybrids in terms of which sequences are retained, pointing to shared stronger constraints. Finally, an article by Heineika and El-Samad looks further back in time by studying the regulatory fate of the Protein Kinase A (PKA) regulon after the ancestral whole genome duplication (WGD) in yeasts. This WGD results from an ancestral hybridization between two divergent non-WGD clades (Marcet-Houben and Gabaldón, 2015)⁠, but few studies take this into account when studying regulatory divergence in ancient paralogs of this clade. By using comparative transcriptomics, Heineika and El-Samad elegantly reconstruct the regulatory evolution of these genes, showing that the different regulatory modes were pre-existing in each of the parental lineages and were brought together by the hybridization event. This study provides perhaps the first evidence that regulatory divergence in hybridizing species may promote ohnolog retention in post-WGD species.

Due to their unique ability to adapt to new niches, hybrids are common among human-altered environments, including industrial settings. The two remaining articles of this Research Topic focus on *Saccharomyces* hybrids of industrial interest. The contribution by Peltier et al. investigates how flor yeasts regulate their central carbon catabolism during wine fermentation. Using quantitative trait locus (QTL) mapping in the progeny of crosses between two wine starter strains, one of which had an admixed genome with flor strains, the authors identify genetic factors of adaptive divergence between the flor yeast and the wine yeast and show also that introgressions from flor strains promoted the metabolic variability observed. Finally, the article by Krogerus et al. use synthetic tetraploid hybrids between *Saccharomyces cerevisiae* and *Saccharomyces eubayanus* to generate genetic diversity in the F2 progeny through meiotic segregation. They show that some of these derived strains show fermentation capacities similar to commercial lager strains and found that higher ploidy was generally associated with faster fermentation. This study highlights the potential of synthetic hybrid formation, followed by selection on the progeny, to generate new strains of industrial relevance.

Altogether, the six contributions to this Research Topic provide important new insights on diverse aspects related to fungal hybrids and underscore the potential of genomic approaches to study them.

## Author contributions

All authors listed have made a substantial, direct, and intellectual contribution to the work and approved it for publication.

## Funding

TG acknowledges support from the Spanish Ministry of Science and Innovation for grant PID2021-126067NB-I00, cofounded by European Regional Development Fund (ERDF); from the Catalan Research Agency (AGAUR) SGR423; from the European Union’s Horizon 2020 research and innovation programme (ERC-2016-724173); from the Gordon and Betty Moore Foundation (Grant GBMF9742); from the “La Caixa” foundation (Grant LCF/PR/HR21/00737), and from the Instituto de Salud Carlos III (IMPACT Grant IMP/00019 and CIBERINFEC CB21/13/00061- ISCIII-SGEFI/ERDF). CTH acknowledges support by the Great Lakes Bioenergy Research Center, U.S. Department of Energy, Office of Science, Office of Biological and Environmental Research under Award Number DE-SC0018409; the National Science Foundation under Grant Nos. DEB-1442148 and DEB-2110403; and the USDA National Institute of Food and Agriculture (Hatch Project 1020204). CTH is an H. I. Romnes Faculty Fellow, supported by the Vice Chancellor for Research and Graduate Education with funding from the Wisconsin Alumni Research Foundation.

## Conflict of interest

The authors declare that the research was conducted in the absence of any commercial or financial relationships that could be construed as a potential conflict of interest.

## Publisher’s note

All claims expressed in this article are solely those of the authors and do not necessarily represent those of their affiliated organizations, or those of the publisher, the editors and the reviewers. Any product that may be evaluated in this article, or claim that may be made by its manufacturer, is not guaranteed or endorsed by the publisher.
